# Beyond morphs: Inter‐individual colour variation despite strong genetic determinism of colour morphs in a wild bird

**DOI:** 10.1111/jeb.14124

**Published:** 2022-12-09

**Authors:** Maya C. Mould, Michèle Huet, Lou Senegas, Borja Milá, Christophe Thébaud, Yann Bourgeois, Alexis S. Chaine

**Affiliations:** ^1^ Station d'Ecologie Théorique et Expérimentale du CNRS and Université Paul Sabatier (Toulouse III) UPR 2001 Moulis France; ^2^ National Museum of Natural Sciences, Spanish National Research Council (CSIC) Madrid Spain; ^3^ Laboratoire Évolution et Diversité Biologique (EDB), UMR 5174 Centre National de la Recherche Scientifique (CNRS), Institut de Recherche pour le Développement (IRD) Université Paul Sabatier (Toulouse III) Toulouse Cedex France; ^4^ School of Biological Sciences University of Portsmouth Portsmouth UK; ^5^ Institute for Advanced Studies in Toulouse Toulouse France

**Keywords:** colour polymorphism, colour quantification, genetic of coloration, morph classification, *Zosterops borbonicus*

## Abstract

Categorizing individuals into discrete forms in colour polymorphic species can overlook more subtle patterns in coloration that can be of functional significance. Thus, quantifying inter‐individual variation in these species at both within‐ and between‐morph levels is critical to understand the evolution of colour polymorphisms. Here we present analyses of inter‐individual colour variation in the Reunion grey white‐eye (*Zosterops borbonicus*), a colour polymorphic wild bird endemic to the island of Reunion in which all highland populations contain two sympatric colour morphs, with birds showing predominantly grey or brown plumage, respectively. We first quantified colour variation across multiple body areas by using a continuous plumage colour score to assess variation in brown‐grey coloration as well as smaller scale variation in light patches. To examine the possible causes of among‐individual variation, we tested if colour variation in plumage component elements could be explained by genotypes at two markers near a major‐effect locus previously related to back coloration in this species, and by other factors such as age, sex and body condition. Overall, grey‐brown coloration was largely determined by genetic factors and was best described by three distinct clusters that were associated to genotypic classes (homozygotes and heterozygote), with no effect of age or sex, whereas variation in smaller light patches was primarily related to age and sex. Our results highlight the importance of characterizing subtle plumage variation beyond morph categories that are readily observable since multiple patterns of colour variation may be driven by different mechanisms, have different functions and will likely respond in different ways to selection.

## Graphical abstract

Inter‐individual color variation in a polymorphic wild bird endemic to the island of Reunion (*Zosterops borbonicus*). Our results highlight the importance of characterizing subtle plumage variation beyond morph categories.

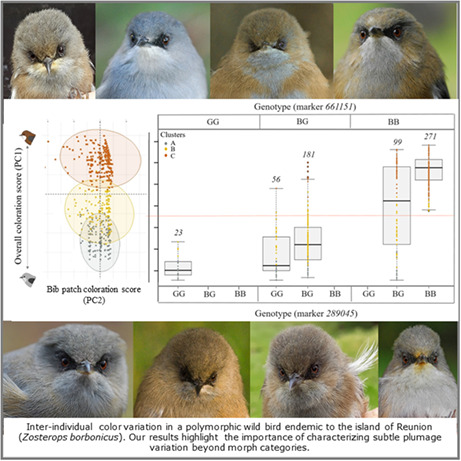

## 1 INTRODUCTION

Colour polymorphism implies the co‐occurrence of discrete morphs by definition, but the extent to which variation between individuals can fit into discrete classes or is continuous with a strongly bimodal (or multimodal) distribution has recently been a subject of debate (Cote et al., 2008; Davison et al., 2019; Paterson & Blouin‐Demers, 2017; Vercken et al., 2008). Studies of colour polymorphic systems have often focused on specific colour patches that best separate morphs to classify individuals into discrete categories rather than integrating colour variation across the entire body (but see Chaine & Lyon, 2008, 2015; Greene et al., 2000). Although some species seem to show a clear pattern of discrete polymorphism (Pryke & Griffith, 2006; Teasdale et al., 2013), some recent studies on classic cases of colour polymorphism have shown that variation among individuals may be better described by a continuous distribution owing to extensive within‐morph variation or the presence of intermediate types (Brommer et al., 2005; Davison et al., 2019; Parejo et al., 2018; Paterson & Blouin‐Demers, 2017; Rankin et al., 2016). Categorization of individuals based on one or two specific patches simplifies measures in the field and analyses, but runs the risk of ignoring additional variation, which could provide important insight into the proximate basis of inter‐individual phenotypic variation and the evolutionary processes that maintain such variation.

Quantitative colour variation within morphs is often associated with condition dependence or phenotypic plasticity (Hill & Montgomerie, 1994), but it also can result from the genetic architecture of the polymorphism itself (Kappers et al., 2018). For example, codominance between two or more alleles on a single locus may lead to a higher diversity of phenotypes than expected under simple dominance models (O'Neill & Beard, 2010; Sinervo et al., 2001). Similarly, while much between‐morph variation could be controlled by one or a few major‐effect loci, several additional loci with minor effects could also contribute to variation among individuals within recognized morphs (Phillips, 2008). Descriptions of continuous variation in colour within and among morphs are, therefore, essential to understand the genetic determinism of colour and downstream consequences of such proximate factors.

Accounting for inter‐individual variation within and among morphs of a species could also improve our understanding of evolutionary processes such as the maintenance of polymorphism and selection on multiple traits. Morphs often covary with other traits that might be under selection, including life history (Pryke et al., 2012), behaviour (Küpper et al., 2016; Sinervo et al., 2006) and physiological traits (Mills et al., 2008). Different variants could correspond to several optimal combinations of traits that are under selection (i.e. correlational selection), each corresponding to alternative peaks across the fitness landscape (Sinervo et al., 2001; Willink et al., 2013), maintained under balancing selection or under spatio‐temporal variation in selection. Frequency dependent selection could also favour the coexistence of multiple colour variants, if rare variants are at an advantage with regards to predator avoidance (Torres‐Dowdall et al., 2017) or sexual and social selection (Dijkstra et al., 2010; Hughes et al., 2013). Likewise, social selection can maintain extensive colour variation through advantages of signalling subtle variation in information (Chaine et al., 2011; Kraaijeveld et al., 2004). Therefore, measuring variation in colour patterns in polymorphic species is a first step that, when combined with information on fitness, could provide important information about the evolutionary mechanisms operating in these systems.

Here, we examined a striking melanin‐based plumage colour polymorphism in natural populations of the Reunion grey white‐eye (*Zosterops borbonicus*), a textbook example of intraspecific variation in plumage coloration in birds (Gill et al., 2019). This species, endemic to the island of Reunion (south‐western Indian Ocean), consists of four distinct adjacent and geographically non‐overlapping forms that exhibit discrete differences in morphology and plumage and have extremely narrow hybrid zones where they meet (Delahaie et al., 2017; Gabrielli et al., 2020; Gill, 1973; Milá et al., 2010). Three forms, restricted to the lowlands, differ strikingly in plumage colour with no documented variation within each form, whereas a fourth form, restricted to the highlands, comprises two very distinct colour morphs with no apparent sexual dimorphism or age‐structured variation (Bourgeois, 2013; Gill, 1973). Birds show predominantly grey or brown plumage, respectively, depending on eumelanin/phaeomelanin deposition (Bourgeois et al., 2017; Cornuault et al., 2015; Gill, 1973). In a recent genetic analysis of this colour polymorphism, Bourgeois et al. (2017) showed that differential deposition of phaeomelanin on back feathers appears to be controlled by a simple genetic mechanism involving a large‐effect locus, with a recessive ‘brown’ and a dominant ‘grey’ allele. Despite this dimorphism in back colour, there is substantial variation in the colour of other major body parts (e.g. auricular, flank and underparts) that are not always consistent with morph classification based on back colour (Cornuault et al., 2015; Gill, 1973). Furthermore, two light patches (grey or white feathers that contrast with surrounding plumage) are characterized by high inter‐individual variability and could be independent from the overall grey/brown coloration. Such variation among individuals within morphs suggests that the genetic mechanisms underlying plumage colour variation as a whole are more complex than the simple Mendelian inheritance mechanism that has been proposed for back colour (Bourgeois et al., 2017). Both the nature of the genetic mechanisms underlying colour and the existence of distinct colour patches could have important consequences for how evolution acts on such traits.

We assessed inter‐individual variation in plumage coloration among 751 individuals by using combined colour scores from nine distinct colour patches distributed across the entire body to allow a thorough and comprehensive characterization of each individual's colour phenotype. We asked if variation among individuals aligned with the two colour morph categories from previous studies and examined the extent of variation among individuals both within and among morphs. We then examined the proximate basis of among‐individual variation in coloration by using two complimentary approaches. Using two molecular markers (Single‐nucleotide polymorphisms; SNPs) positioned near the causal major‐effect locus for back colour, we examined to what extent these SNP markers influence variation in plumage coloration across multiple body areas both within and among morphs. In addition, we investigated other factors that could influence colour phenotype such as age, sex and condition, which are often related to variation in colour (Hill & Montgomerie, 1994; Kraaijeveld et al., 2004; Lyon & Montgomerie, 1986). Taken together, this study should help identify the extent of colour variation in a classic example of colour polymorphism, allow a better understanding of the proximate control of colour through the relationship between colour and genetic markers and sets the stage for further investigations into the selective forces that maintain colour polymorphism across time when combined with temporal frequency or fitness data.

## 2 MATERIAL AND METHODS

### 2.1 Fieldwork and data collection

Fieldwork was carried out at three sites on the Piton de la Fournaise volcano: Nez de Boeuf (−21.205726, 55.618666), Pas de Bellecombe (−21.217345, 55.687544) and Bois Ozoux (−21.197549, 55.646322), located, respectively, at 2070, 2246 and 2283 meters above sea level. Birds were captured using mist nets in April–May (non‐breeding season) from 2007 to 2019 as well as in October 2018 (breeding season) and immediately released after data collection at the same location. Each bird was ringed with a numbered aluminium ring provided by the CRBPO. We measured tarsus length to 0.1 mm and body mass to 0.1 g.

We collected ~10 μL of blood for genetic analysis at the first capture of each bird by puncturing the sub‐brachial vein and collecting blood with a capillary tube. Blood was conserved in Queen's lysis buffer for samples prior to 2014, or ethanol for those after 2014 and stored at −20°C for long‐term preservation. We extracted DNA using QIAGEN DNEasy Blood and Tissue kits following manufacturer instructions.

We photographed birds at capture from four different angles (back, front, ventral and lateral) in the shade and against a uniform background for colour classification (see below). From 2018, a ruler was added next to the bird to measure colour patch surface area from images. Birds were categorized in the hand as brown or grey depending on whether they showed predominantly brown or grey plumage (Bourgeois et al., 2017; Cornuault et al., 2015; Milá et al., 2010). These birds included 358 brown and 317 grey individuals as well as 45 individuals that could not be clearly assigned to either morph and 31 with missing assignment. Over 12 years, we caught a total of 751 birds with 179 individuals recaptured at least once (up to 11 years later).

### 2.2 Plumage coloration score

**FIGURE 1:**
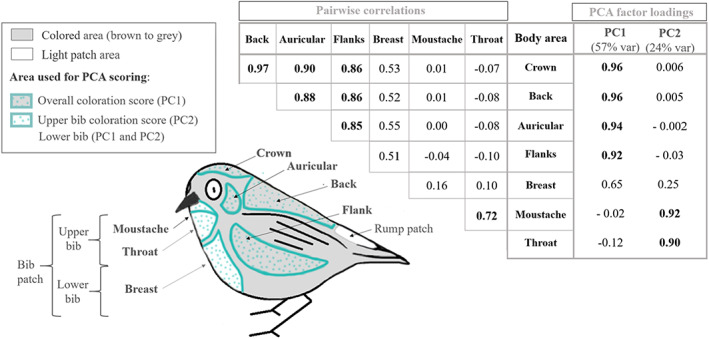
Pairwise correlations and factor loadings onto the first two principal component axes for each plumage region included in coloration scores. Corresponding plumage areas that were scored and included in the PCA analysis are represented on the bird with areas surrounded and spotted in blue.

We described variation in plumage using a human visual colour score based on photos taken in the field because the colour traits of interest reflect only wavelengths within the human visual range (Bourgeois et al., 2017; Cornuault et al., 2015) and because plumage variation was mainly characterized by the presence/absence of particular patterns of coloration rather than by intensity of the coloration itself (Montgomerie, 2006). Scoring was performed by only one person to ensure consistent application of the scoring scale, which was adapted from colour indexes used by Gill (1973). For each of seven key body areas (back, crown, auricular, flank, moustache, throat and breast) (Figure 1), a score ranging from 0 (white) or 1 (grey) to 7 (full brown) was attributed to describe the extent of grey and brown feathers within the area. Rump patches were white and did not vary in colour, so we did not include in colour scoring (see below for patch size estimates).

We collapsed variation in scores for the seven body areas using a Principal Components Analysis (PCA) and extracted values of each principal axis to describe an individual's unique overall colour score (e.g. Chaine & Lyon, 2008, 2015). PCA was performed using the package *ade4* (Chessel et al., 2004) in R 3.0.2 software (R Core Team, 2018). We retained the first two PCA axes with eigenvalues of 4.03 and 1.73, respectively, which captured considerable variation in plumage colour for each of the 7 different body regions (Figure 1). The first axis (PC1) explained 57% of the variance in plumage regions and described variation in the back, crown, auricular and flank (Figure 1, Figure S1), which are the most visible areas of the body (Figure 1). Thus, PC1 represented the overall body coloration and ranged from −3 (full grey phenotype) to +3 (full brown phenotype) and is hereafter called ‘overall coloration score’. The second principal axis (PC2) explained 24% of the variance in plumage colour and described variation in moustache and throat coloration (corresponding to the upper part of the bib patch (Figure 1)) and ranged from −2 (white) to +3 (brown) (Figure S1). These scores are hereafter called ‘upper bib coloration scores’. Variation in breast coloration (which corresponds to the ‘lower’ part of the bib patch (Figure 1)), loaded somewhat on both axes although more so on PC1 than PC2 (Figure 1).

Repeatability of scoring colour was determined using 54 random pictures that were scored twice by the same person one year after initial scoring. Because we did not have repeated photos of the same bird captured more than once within a year, we could not test repeatability on different pictures. The repeatability was high (*R* = 0.95), suggesting the scoring method is robust (package *rptR* (Stoffel et al., 2017)). Moreover, we also estimated colour score repeatability on birds caught and photographed several times across years (179 individuals) and despite this time delay in which plumage could change as a result of moult, repeatability was high (*R* = 0.89) suggesting that plumage colour did not change over time.

To determine if our scoring method showed discrete morphs as in previous work, we performed cluster analysis using raw colour scores of the back, crown, auricular and flank (without moustache, throat, breast that did not load on our overall coloration score). We used a Hierarchical Agglomerative Clustering (HAC) analysis based on the ‘ward.D2’ method to assign individuals to clusters (package *stats*; Figure S2). To identify the number of clusters that best fits our data, we ran HAC for 2–10 clusters, assigned individuals to the defined number of clusters and then calculated metrics of fit for each defined number of clusters using the *NbClust* package (Charrad et al., 2014). We calculated 24 indices (Table S2) of fit provided in *NbClust* and followed the majority rule to determine which number of clusters best fit our data. We then compared continuous colour scores along the two major PCA axes (overall coloration and bib coloration) against both discrete morph classification in the field into the two grey/brown categories and the three categories identified in the HAC analysis (Figure 3).

### 2.3 Quantification of light patches

We quantified the surface area of two light patches found on the bird's rump and bib using photographs of 300 birds taken in 2018–19 that included a ruler for scale. The rump patch is a clearly delimited white area surrounded by either brown or grey according to the morph (Figure 1), so we measured the surface of this white patch using Photoshop software to calculate the number of pixels in the rump patch and converting it to area in mm^2^ using the number of pixels per mm on the ruler in the same photo (e.g. Chaine et al., 2011; Chaine & Lyon, 2015). Note that white rump patch sizes are only available for a small subset of birds (2018–2019 vs 2007–2019 for other colour traits) in the coloration dataset and so was not included in compilation of PC colour scores.

**FIGURE 2:**
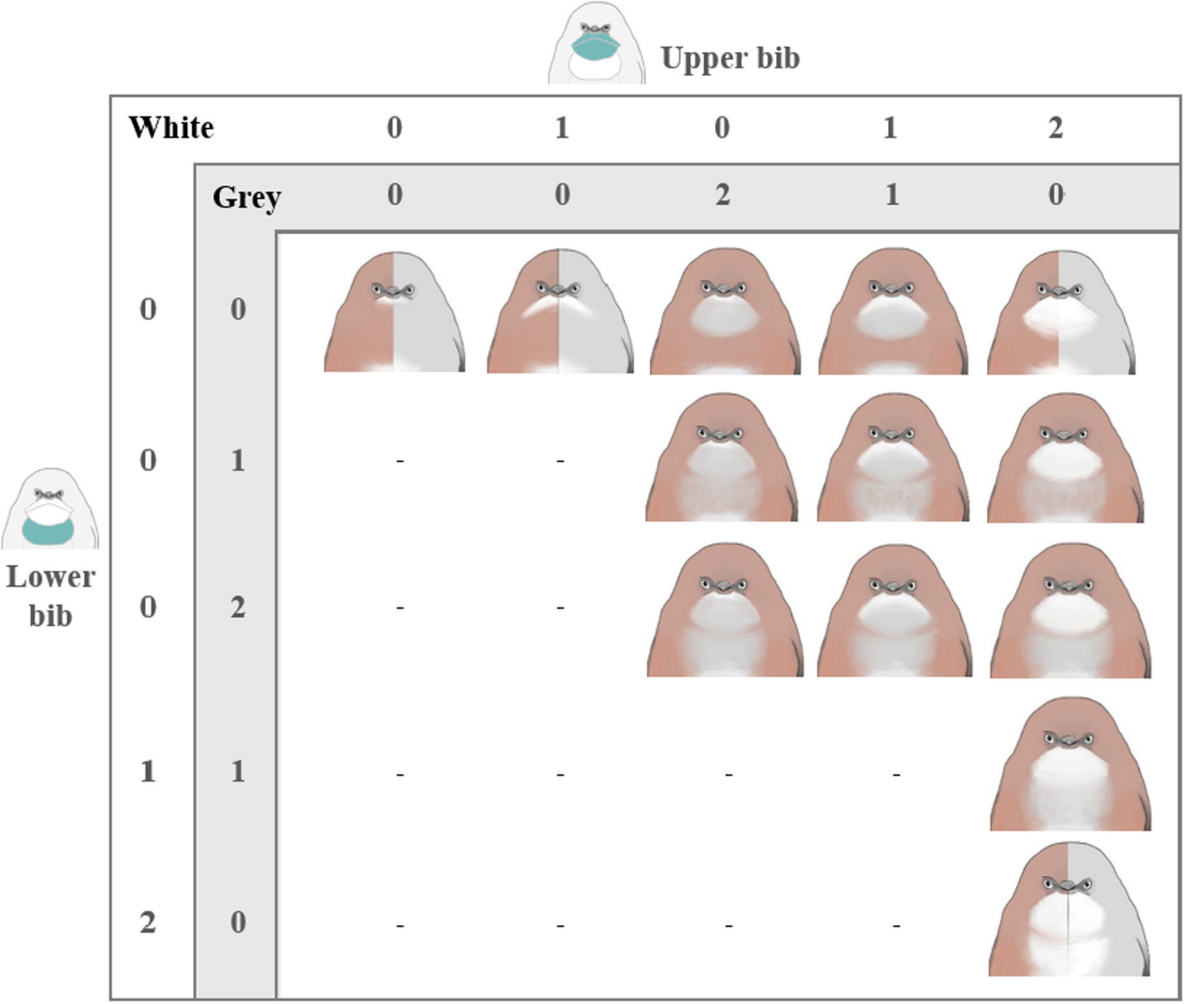
Classification of bib patches is described in two separate scales by the extent of white feathers and the extent of grey feathers for both the upper bib (rows, in yellow) and the lower bib (columns, in red). Bib patch scores result from the addition of white and grey scores for upper bib coverage, lower bib coverage, white coverage (addition of white scores on both upper and lower bibs) and grey coverage (addition of grey scores on both upper and lower bibs). For example, the last class at the bottom right of the table has a total score of 4 (larger size), with an upper bib and a lower bib surface of 2 each, a white score of 4 and a grey score of 0 (i.e. full white patch). Combinations that do not exist are noted with ‘–’ The four types that exist both for grey and brown morphs are represented by birds divided in their coloration. The other nine brown specific types do not exist in grey birds since a grey area surrounded by grey plumage was not a visible patch.

The light area composing the bib patch (below the bill to the belly) ranges from white to grey and is, therefore, difficult to delineate because grey coloration of the body can be confounded with grey coloration of the patch. As such, we categorized bib area using patch surface as a proxy of size, based on the presence/absence of light areas on the bib patch relative to overall coloration (see Figure 1). Bib patch analysis was performed on photos of 583 birds caught from 2007 to 2019. This classification relied on the combination of two parameters: (i) the coverage of each of the two major areas that make the bib patch (‘upper bib’ and ‘lower bib’ (Figure 1)) and their addition named ‘total bib coverage’; and (ii) the coloration of the patches (white or grey) (Figure 2). The three bib patch coverage measures (i.e. upper, lower and total bib) showed similar results, so we present results on total bib coverage and white versus grey coverage within the main text and upper and lower bib coverage results in Table S3.

We used linear mixed models to test for a relationship between plumage components (overall coloration score, bib patch white, grey and total coverage and rump patch size). We did not consider the relation between the overall coloration and the upper bib coloration score, which by definition are uncorrelated (orthogonal PCA axes). Morph frequencies vary between sites and years (C. Thébaud, B. Milá, M.C. Mould, unpublished data) and some plumage components ‐ in particular those highly variable among individuals ‐ could be influenced by environmental effects, so we included site and year as random effects. We also included bird identity as a random effect.

### 2.4 Influence of the major‐effect colour locus on between‐ and within‐morph plumage colour variation

To test for an association between the major‐effect locus and plumage variation measured using our new continuous colour score, we conducted a molecular survey at two diagnostic SNP markers (named 289 045 and 661 151) separated by 180 kb and situated, respectively, at position 2 412 623 and 2 589 332 on scaffold 40 of the *Zosterops lateralis* reference genome (Figure S3). Data were obtained from a pooled RAD‐sequencing experiment performed by Bourgeois et al. (2017) (European Nucleotide Archive repositories: accession n° ERP020509 and ERP002555; BioProject PRJEB18566 available at https://www.ebi.ac.uk/ena/browser/view/PRJEB18566?show=reads). Scaffold 40 originates from the *Zosterops lateralis* reference genome, which aligns to the Zebra Finch chromosome 1 (available at http://m.ensembl.org/Zosterops_lateralis_melanops/Info/Annotation#page_nav; Bourgeois et al., 2017). These markers encompass a region with a high density of SNPs that are strongly associated with differences in back colour. They are likely located close to the causal locus that determines back coloration according to past work (Bourgeois, 2013; Bourgeois et al., 2017). We genotyped each marker separately using a Single Specific Primer‐PCR approach, using optimized conditions (Table S1). We visualized PCR products of each allele separately on 1% agarose gels. Each allele at a marker was run separately to have the corresponding bands either for the brown allele or grey allele. Brown homozygotes showed a band on the gel loaded with the brown‐allele primer, but none on the gel with the grey‐allele primer, whereas grey homozygotes had the reverse, and heterozygotes had a band on both gels.

In Bourgeouis et al.'s (2017) analysis, the causal locus was unidentified but close and strongly linked to the two SNP markers we scanned and possibly located upstream of the selective sweep (Figure S3). Bourgeois's analysis suggests that marker 661 151 should be more tightly linked to the causal locus than marker 289 045 (Table S1), and recombination tests show that both markers are strongly but incompletely linked to each other, therefore, we should expect more recombination events between the causal locus and SNP marker 289 045 than between the causal locus and SNP marker 661 151. In addition, there might be modifiers between the causal variant and each SNP marker, a few double recombinants, or genotyping errors at one SNP marker, which could all explain some additional variation in phenotypes relative to genotypes. As such, looking at a single SNP marker can miss the actual genotype at the causal locus, while looking at both improves our power to understand the importance of the putative causal locus in determining colour variation in this species. The combination of the two SNP markers is hereafter presented in the order marker 289 045/marker 661 151, with allele B corresponding to the brown phenotype allele and G to the grey phenotype allele (for example BGBB is heterozygote on marker 289 045 and brown homozygote on marker 661 151). We estimated linkage disequilibrium between the two markers using R package genetics (Warnes, 2011) as well as the partial correlation coefficient of each marker in relation to the overall coloration score in order to understand the relationship of each marker with the colour phenotype. We tested the relationship between SNPs and our overall coloration score, light patch coloration and bib surface score, using a linear mixed model with the full genotypes (combination of both markers). Using the same method, we also estimated the relationship between the two SNP markers and each plumage area independently (Table S4).

### 2.5 Effect of sex, age and body condition

We determined sex using sex‐chromosome specific markers for the *CHD1* gene (Griffiths et al., 1998) following standard PCR conditions (Table S1) visualized on 1% agarose gels. Age was assessed in the field and classified as adult (after first year), juvenile (first year), or fledglings (<3 months after fledgling) based on moult characteristics, skull ossification and iris colour (i.e. pale greyish‐orange in juveniles and darker reddish‐brown in adults). A proxy of body condition, ‘residual mass’, was calculated using the residuals of body mass regressed on tarsus length (Schulte‐Hostedde et al., 2005) in models including ‘ringer ID’ as a random effect to account for differences in measurement between ringers.

To understand how individual characteristics might be related to plumage, we conducted linear mixed effects models to examine the influence of age, sex and body condition on (i) overall coloration scores (PC1), (ii) upper bib coloration scores (PC2), (iii) bib patch surface (total bib coverage, white coverage and grey coverage) and (iv) rump patch area. We included the interaction between age and sex in our models as sexes sometimes differ in the development of adult plumage (Lyon & Montgomerie, 1986). We included sites and the interaction between site and body condition in our model because variation in morph frequency and body condition could be site‐dependent. Overall coloration score was included in models predicting bib and rump patch scores in order to understand the relationship between individual characteristics and colour patches while controlling for overall plumage colour. Year of capture and bird identity were included as random effects in all models.

## 3 RESULTS

### 3.1 Variation in plumage components

**FIGURE 3:**
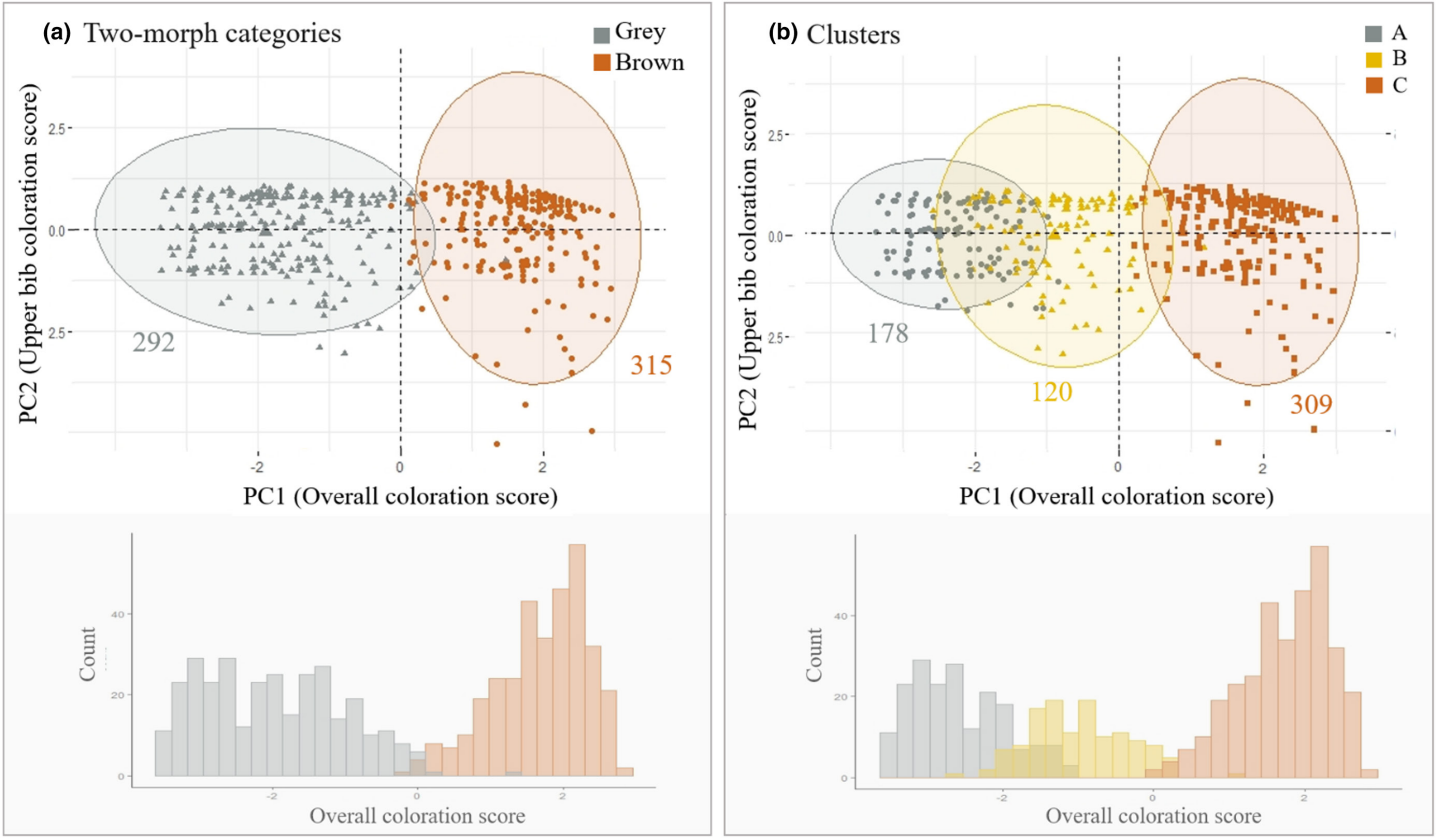
Comparisons of continuous plumage coloration scores according to morph classification into (a) 2 categories (grey vs brown) based on back coloration versus (b) 3 categories (grey, tan, brown) identified in HAC analysis of scores at each colour patch. Upper plots show among‐individual variation in the two PCA scores summarizing colour variation for overall body coloration (PC1) and upper bib coloration (PC2). Ellipses encompass individuals assigned to each morph category. Lower plots show histograms of the number of individuals by continuous body coloration score.

Hierarchical agglomerative clustering (HAC) of individual colour patch scores identified three major groups (Figure S2, Table S2) according to the majority rule of the 24 indices we calculated for 2–10 clusters (see Table S2). The clustering dendrogram shows a first major division between largely brown birds (Group C) and the other two groups, which include a pure grey cluster (Group A) and intermediate birds that were overall grey but with an appreciable number of brown feathers mixed in (Group B; Figure S2). The frequencies of the different phenotypes along the continuous overall coloration score showed a clear multimodal distribution with two strong modes corresponding to the brown morph and to the grey morph and another smaller mode corresponding to intermediates identified as group B in HAC (Figure 3b, Figure S2). The separation of the two currently recognized morphs into three groups identified in HAC occurred along the first PCA axis corresponding to overall coloration with no separation along the second PCA axis corresponding to bib patches. The brown morph largely corresponds to brown birds in Group C identified by HAC (315 brown individuals grouped by morph in Figure 3a, versus 309 grouped by cluster in Figure 3b). In contrast, birds classified as grey morph in the field showed more variation along the first principal axis corresponding to overall coloration in our continuous colour scores. The cluster analysis separated these birds into two groups (Figure 3a vs Figure 3b) corresponding to pure grey (Group A, 178 birds) and intermediate birds that are predominantly grey but with some brown feathers (Group B, 120 birds) (Figure 3b). Finally, there was a continuity in overall colour score between clusters, with considerable overlap between the pure grey and intermediate categories (Figure 3b). High variance in overall colour within clusters and overlap in the coloration of birds in each cluster suggests that there is important intra‐morph variation in coloration (Figure 3b).

We asked to what degree different plumage components (i.e. overall body coloration, bib and rump patch) were correlated. Overall coloration of an individual (brown to grey) had an impact on the visibility of the bib patch, while controlling for site, year of capture and band number as random effects. The white part of the bib area was unrelated to bird's overall coloration score (white coverage: F_1,502_ = 1.95; *p* = 0.16). In contrast, the grey bib area was only visible in brown birds, making its size larger in brown birds (grey coverage: F_1,489_ = 93.13; *p* > 0.001). Consequently, brown individuals had a larger total bib coverage, which includes both white and grey areas (F_1,487_ = 120.12; *p* > 0.001). Conversely, grey coloured individuals had a larger rump patch (*r* = − 0.011; F_1,223_ = 5.06; *p* = 0.02). The surfaces of the two light patches, bib and rump, were not related to each other (F_1,217_ = 0.07; *p* = 0.78).

### 3.2 Association between genetic markers and colour phenotypes

**FIGURE 4:**
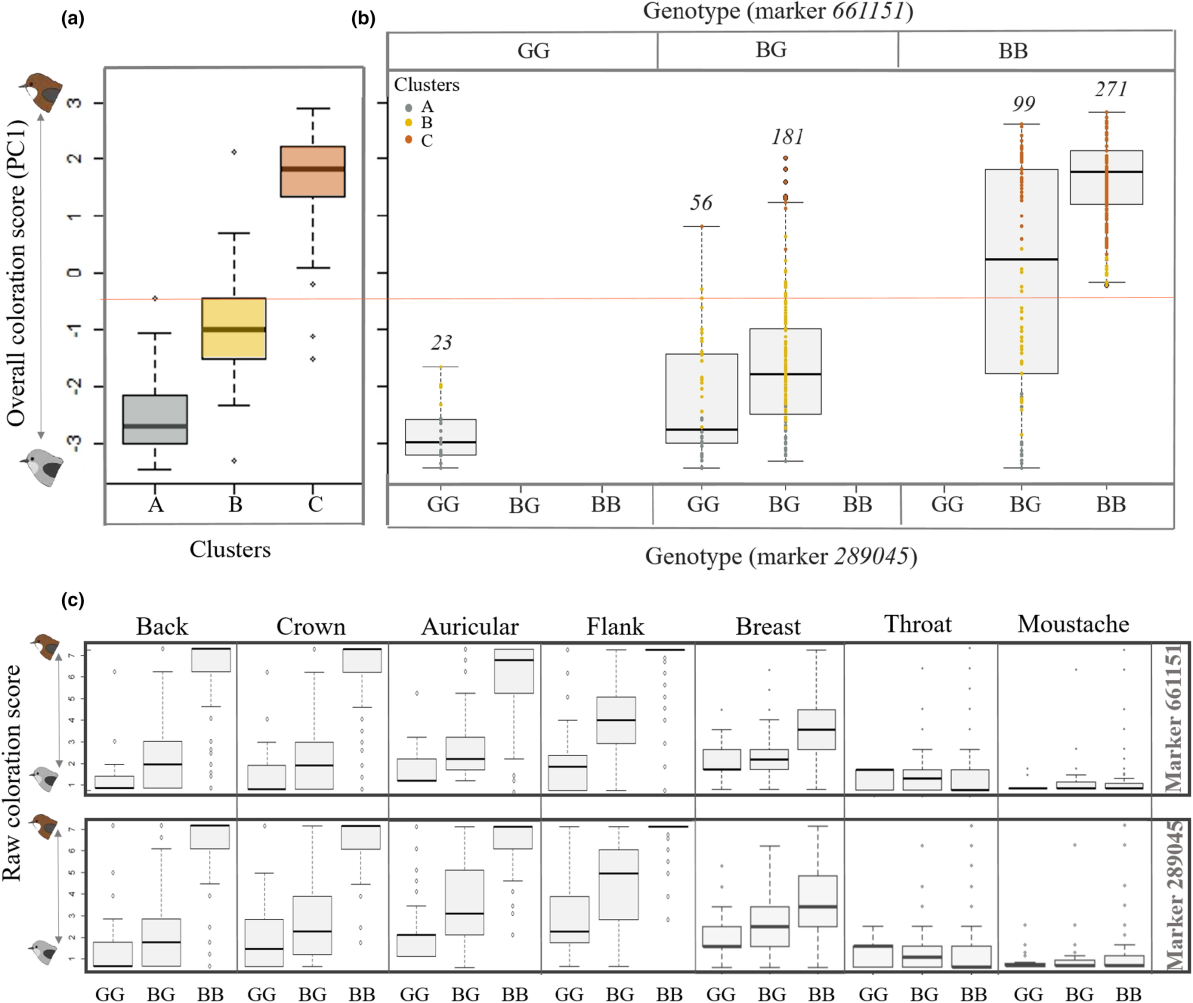
Comparisons of the overall plumage coloration score between a) the three colour morph clusters (‘A’, ‘B’ and ‘C’) identified by HAC analysis and b) the five observed genotypic classes. Colours for individual data points correspond to the three categories identified by HAC as in panel (a). The red line represents the midpoint (= − 0.58) between the mean colour scores for the A and C clusters. In (c), distribution of coloration for each genotype at each body area. The first four body areas listed (back, crown, auricular and flank) loaded on the first PCA axis (overall coloration score), whereas the last two (moustache and throat) loaded on the second PCA axis. Raw coloration scores go from 0 (white) or 1 (grey) to 7 (brown), with all the intermediate scoring between extreme grey and extreme brown. Genotypes are represented for marker 661 151 in upper panels and for marker 289 045 in lower box panels. B is the brown allele and G is the grey allele. Relationship between PC2 and genotypes are in supplemental Figure S4.

The combined effect of genotypes at both SNP markers explained 68% of the variation in the overall coloration score (PC1) (F_4,578_ = 315.6; *p* < 0.001), but had no influence on the coloration or size of light patches (upper bib coloration scores (PC2): F_4,547_ = 0.65; *p* = 0.6, total bib coverage: F_4,378_ = 0.66; *p* = 0.61, rump patch size: F_4,177_ = 0.88; *p* = 0.47) (Figure S4). Marker 289 045 and marker 661 151 explained 36% and 40% of the overall coloration score (partial correlation coefficient), respectively (no interaction effect: F_3, 551_ = 2.2; *p* = 0.08) and showed high but incomplete linkage disequilibrium (D = 0.14; D′ = 0.97; Corr = 0.74). The combination of the genotypes at the two markers provides information on both recombination between the two markers and genotyping errors. While the majority of combined genotypes were composed of the same genotypes at the two markers (75% of genotypes were either BBBB, BGBG or GGGG), 25% had different genotypes at the two markers (GGBG and BGBB) (Figure 4b), suggesting there were substantial levels of recombination between them. Since SNP marker 661 151 should be more linked to the causal locus than SNP marker 289 045 (Table S1), we expect more recombination events between the causal locus and marker 289 045 than between the causal locus and marker 661 151. In addition, there might be modifiers between the causal variant and each marker, which could explain some additional variation we observe in phenotypes. As such, we can assume the GGGG, BGBG and BBBB are most likely GG, BG and BB, respectively, at the causal locus, but cannot predict genotypes at this putative causal locus with confidence for GGBG and BGBB individuals. Interestingly, some single recombinant genotypes (BBBG and GBGG) and double recombinant genotypes (GGBB and BBGG) at the SNP markers could exist in theory, but were never seen in our data.

We found that heterozygotes (BGBG) had a higher mean overall coloration score (= − 1.61, i.e. browner) than grey homozygotes (= − 2.83), but much lower than would be expected under full codominance (= − 0.58; red line in Figure 4), suggesting partial dominance of the grey allele over the brown allele. This pattern was true for all parts of the grey/brown body coloration (Figure 4c). The similarity in overall colour distributions of individuals identified in each cluster by HAC with genotypic categories suggests that intermediate individuals that are predominantly grey but with appreciable number of brown feathers mixed in are likely heterozygotes (Figure 4a,b,c). Indeed, grey homozygotes fell almost exclusively in cluster A whereas brown homozygotes fell into cluster C (Table 1). While cluster B was composed nearly exclusively of heterozygotes (only 4% grey and 3% brown homozygotes were assigned to the cluster B; Table 1), many heterozygotes were also assigned to cluster A (grey) (Table 1).

**TABLE 1:** Proportion of individuals at the five combined genotypic classes (marker 289 045/marker 661 151) according to their classification in cluster a (grey), B (intermediate) and C (brown)

Cluster	GGGG	GGBG	BGBG	BGBB	BBBB
A	0.96	0.73	0.51	0.28	0.00
B	0.04	0.25	0.44	0.23	0.03
C	0.00	0.02	0.05	0.49	0.97

*Note*: Recombinant genotype columns are shown in grey as they are likely composed of both homozygotes and heterozygotes at the causal locus, whereas GGGG, BGBG, BBBB are most likely GG, BG and BB at the causal locus.

### 3.3 Influence of sex, age and condition on plumage coloration

We found that overall coloration did not differ between the sexes (F_1,386_ = 1.31; *p* = 0.25) nor between age categories (chick, juvenile, adult: F_2,176_ = 0.34; *p* = 0.71) or their interaction (age×sex F_2,250_ = 1.05; *p* = 0.35) confirming that there is no sexual dimorphism or age‐related variation for overall coloration. Brown individuals were in better body condition than grey individuals (*r* = 0.024; F_1,172_ = 3.91; *p* = 0.04) and this relationship did not differ by site (site×condition F_2,251_ = 1.98; *p* = 0.14).

Males had larger overall bib patches than females (total bib coverage: F_1,272_ = 10.19; *p* = 0.001), which occurred because males had a larger white bib area coverage (white bib area: F_1,278_ = 17.15; *p* < 0.001) when bibs were white, whereas bib area was similar between the sexes when bibs were grey (grey bib area: F_1,286_ = 0.04; *p* = 0.84). Likewise, males had a lighter upper bib patch (upper bib coloration score: F_1,381_ = 17.23; *p* > 0.001). Males had lighter upper bib patches earlier in life than females (age×sex interaction: F_2,361_ = 6.86; *p* = 0.001). In general, the size and coloration of bib patches changed with age as the bib patch became lighter (F_2,194_ = 97.51; *p* < 0.001) and larger (total bib coverage: F_2,141_ = 16.66; *p* > 0.001) with age. This was particularly true for the grey area (F_2,198_ = 4.41; *p* = 0.01) since the relation between white area and age was marginal but not significant (F_2,207_ = 2.68; *p* = 0.07). However, most of the changes in size and coloration in bib patches happened between the ‘fledgling’ and ‘first year’ age classes (*t* = 5.63; *p* < 0.001) since very little change was seen between ‘first year’ and ‘adults’ (*t* = 0.41; *p* = 0.67). Variation in rump patch size was not related to age (F_2,141_ = 1.69; *p* = 0.18) or sex (F_1,150_ = 0.17; *p* = 0.67).

There was an interaction between body condition and site on total bib coverage (F_2,287_ = 9.60, *p* < 0.001) and white coverage (F_2,313_ = 0.45; P = 0.04), but not on grey coverage (F_2,314_ = 0.48; *p* = 0.14) nor with the upper bib coloration score (F_2,380_ = 0.30; *p* = 0.73). Variation in rump patch size was not related to body condition (F_1,74_ = 0.24; *p* = 0.62) and there was no interaction between body condition and site (F_2,78_ = 0.57; *p* = 0.56).

## 4 DISCUSSION

Highland populations of the Reunion grey white‐eye provide an intriguing case of apparently stable genetic colour polymorphism with two distinct morphs largely classified by brown or grey back coloration. Past work on this plumage dimorphism focused on back colour (e.g. Bourgeois et al., 2017; but see Gill, 1973 and Cornuault et al., 2015), and here, we expanded our analysis to include other body regions including the colour and size of light‐coloured patches on the throat and rump. Colour scoring of the plumage patches largely fell along two orthogonal axes corresponding to overall body colour (i.e. brown/grey coloration on much of the body) and upper bib patch colour. Interestingly, the bib patch colour and the overall coloration were unrelated, suggesting they might be determined by different mechanisms and have different functions (e.g. Chaine et al., 2011; Pryke & Griffith, 2006; Rathbun et al., 2015). While the colour of the bib patch did not differ among morphs, bib patch visibility was influenced by the morph in part because only white, not grey, patches were visible in grey individuals whereas both white and grey bib patches were visible in brown individuals. Colour morphs are frequently associated with morphological and behavioural syndromes (Küpper et al., 2016; Rathbun et al., 2015; Roulin, 2006; Sinervo et al., 2006), but the three plumage components we identified appear to be somewhat independent and may function as multiple signals (Chaine et al., 2011; Chaine & Lyon, 2015; Nam et al., 2016; Rathbun et al., 2015) that could experience different evolutionary constraints. Multiple colour patches have been highlighted in studies of social communication (Chaine et al., 2018; Marchetti, 1998; Nam et al., 2016), but have rarely been investigated in polymorphic species limiting our understanding of the frequency and function of such signals in polymorphic systems.

Population‐based genome‐wide association studies of plumage colour variation in *Z. borbonicus* have suggested a strong selective advantage for a dominant ‘grey’ allele once it arose from a new mutation, leading to its fast spread across all highland populations (Bourgeois et al., 2017). Since the two recognized morphs that were classified as predominantly grey and brown (Gill, 1973) are found at an appreciable frequency in all populations, this implies that the selected allele does not go to fixation, but instead reaches an intermediate frequency, as would be expected under balancing selection. Our findings are in line with these results, since birds that were classified as intermediate were heterozygous grey individuals with scattered traces of brown pigmentation. Yet, applying continuous colour scales in characterizing colour phenotypes, we also found that beyond discrete morph categories, the different aspects of plumage colour showed previously unsuspected levels of complexity with a rich diversity in multiple plumage colour components. By quantifying colour with continuous scores, we identified a third phenotypic category largely composed of birds previously classified as grey morphs, which were primarily heterozygotes at the two markers linked to the causal locus. Individuals of this intermediate phenotype had a higher coloration score (i.e. were browner) than grey homozygotes, but seemed to be less brown than expected under codominance. Indeed, accounting for such complexity could improve our understanding of how selection acts on polymorphism by understanding how genetic architecture of colour production influences a response to selection and through more accurate measures of selection on alternative alleles to distinguish between heterozygote advantage or negative frequency dependent selection. For example, polymorphism in common buzzards *Buteo buteo* was long described as a simple codominant mendelian system with dark and light morphs and an intermediate morph that appeared to experience a fitness advantage (Krüger et al., 2001). However, recent work has shown that plumage variation is likely polygenic, casting doubt on the hypothesis of a simple heterozygote advantage (Kappers et al., 2018). Similarly, the use of a continuous colour scale in Reunion grey white eyes allowed us to use inter‐individual variation in plumage colour to understand the underlying proximate basis of colour and measure how selection might promote the coexistence of alternative colour phenotypes in this system.

Another benefit of examining continuous variation in plumage coloration beyond discrete morph categories is that we can start disentangling the effects of genetic architecture and environmental influences on phenotype. This may in turn provide insights on the evolutionary consequences of trait variation. We found extensive variation in overall coloration within morphs despite a multimodal distribution with clear peaks around each morph (Figure 3a,b). While our results confirm that a gene of large effect is largely responsible for between‐morph coloration (see discussion below) (Bourgeois et al., 2017), within‐morph variation suggests that additional genetic or environmental factors likely contribute to minor variation in localized body areas that could be of functional significance. The evidence for substantial within‐morph variation in other polymorphic systems is mixed. For example, among‐individual variation appears limited in side‐blotched lizards (Sinervo et al., 2001) and gouldian finches (Pryke & Griffith, 2006). In contrast, recent work in barn owls previously categorized as light or dark found extensive inter‐individual variation in colour (Antoniazza et al., 2010). Likewise, ruffs have extensive within‐morph variation that is thought to be important in individual identity signalling (Dale et al., 2001), even though a supergene is largely responsible for alternative morph appearances (Küpper et al., 2016). Why polymorphic systems differ in the degree of within and among morph variation may depend in part on the genetic architecture underlying phenotypic expression and/or selective pressures that act on social signalling in those species (Rathbun et al., 2015).

### 4.1 Genetic determinism

To understand the role of genes in determining phenotypic variation in Reunion grey white eyes, we examined the relation between genotypes at two markers that reside close to a locus previously linked to back colour in this species (Bourgeois et al., 2017) with classification along our continuous colour scale. We found that the combination of the two markers explained around 70% of inter‐individual variability in overall coloration. Interestingly, predominantly grey individuals with scattered traces of brown pigmentation within grey patches largely correspond to heterozygotes at the two markers linked to the major‐effect locus for back coloration. Bourgeois et al. (2017) proposed dominance of the grey allele, but our analysis using a continuous colour scale reveals that partial dominance is more likely leading to an important shift in our understanding of the genetics underlying colour in this system, in particular because the partial dominance pattern was true for overall coloration scores (PC1) and for all individual parts of the grey/brown body coloration independently (Figure 4c). Partial dominance in colour determining‐loci could be more common than currently described, but would require re‐evaluation of coloration on a continuous scale in other systems. For example, early studies of the side‐blotched lizard identified three morphs controlled by three alleles at one locus (Sinervo & Lively, 1996), but more recent investigations have identified heterozygotes with intermediate phenotypes (Sinervo et al., 2001). The genetic architecture of colour has implications for how selection drives variation within the population. While codominance is frequently associated with selection that favours intermediate phenotypes or multiple morphs (O'Neill & Beard, 2010), partial dominance has rarely been documented in animals, which may in part be because discreet classification of morphs does not allow us to easily distinguish between codominance and partial dominance.

Although strong genetic linkage was found between the two markers we studied, we also found a substantial proportion of recombinant alleles (9% BGBB, 15% of GGBG). Interestingly, we found that genotypes that included the B‐G haplotype (marker 289 045/marker 661 151) were never observed (BBBG and BGGG) whereas the G‐B haplotype existed in our sample (GGBG and BGBB). Based on Bourgeois et al. (2017), the grey allele at the putative causal locus is the one that swept recently. This means that alleles that were associated with the new variant rose rapidly in frequency due to hitch‐hiking. On the other hand, the brown alleles seem to have retained ancestral variation. Therefore, the history of recombination since the emergence of the polymorphism at the causal locus may not have produced this haplotype if the G‐B recombinant emerged relatively early during or after a selective sweep (Bourgeois et al., 2017) allowing it more time to rise to a detectable frequency than B‐G recombinants.

While much of the variation in coloration was explained by one genetic region (major‐effect locus), it is likely that other factors also drive minor within‐morph variation. Indeed, we found considerable overlap in coloration of the three categories identified through clustering analysis. This overlap could be due to multiple genetic factors contributing to coloration or environmental effects on colour expression. Interestingly while all intermediate phenotypes (cluster C) were heterozygotes, only a half of heterozygotes were classified as intermediates whereas the other half were classified as grey. Bourgeois et al. (2017) suggested there are several candidate genes within the major‐effect colour locus and one or more of these gene may be involved in shaping the observed colour variation. Furthermore, additional genes or modifiers could contribute to the effects of the major‐effect locus in determining colour phenotype.

### 4.2 Covariation between plumage and sex, age and body condition

Variation in plumage of many bird species signal sex and age (Kraaijeveld et al., 2004; Lyon & Montgomerie, 1986) or condition (Hill & Montgomerie, 1994). Such signals are expected to evolve since, in many bird species, the discrimination of sex, age, or condition is essential to social selection. Our results show that variation in overall coloration was very stable across time for individuals since there was no relation with age and high repeatability of colour score among years. Similarly, overall coloration was not related to sex as previously reported by Gill (1973). However, we did find a weak relationship between condition and overall coloration since brown individuals tended to be in better condition than grey ones. A relationship between colour and body condition could result from two different processes, either because condition influences the expression of colour (Hill & Montgomerie, 1994) or because differences in competitive abilities result in one morph generally being in worse condition (Pryke et al., 2012). Our results cannot distinguish between the two processes, although it seems less likely that condition influences coloration since overall coloration is highly consistent across time, whereas condition is not.

In contrast to overall coloration, light patches differed between the sexes and changed with age. Males had lighter and larger bib patches than females and bib patch got larger and lighter with age especially between fledgling and first year birds. This is consistent with some theory that suggest that sexual selection favours brighter coloration in males whereas natural selection favours duller coloration in females and younger individuals (Badyaev & Hill, 2003). Interestingly, this effect was driven by the white feathers in a patch, which suggests that age and sex related information in these traits are similar for each colour morph despite grey bib feathers only being visible in brown individuals. In contrast, rump patches were larger in grey morph individuals but did not vary with age or sex. Taken together, it appears that while overall body coloration is largely determined by morph‐specific alleles, light patches are linked age, sex and possibly condition. Age and sex effects on plumage are often a consequence of shifts or differences in hormone production (e.g. Bókony et al., 2008; McGlothlin et al., 2008) and it would be interesting to see if such processes are responsible for variation in bib and rump patches in grey white eyes. While differences in the mechanisms underlying colour patches are common in many species (Kraaijeveld et al., 2004), it remains unclear if multiple signals are common in colour polymorphic species.

## 5 CONCLUSION

By characterizing subtle plumage variation beyond discrete morph categories, we both reinforced and extended our understanding of colour variation in this species. For example, overall colour showed a multimodal distribution consistent with two main plumage colour phenotypes linked to a genetic region of major effect, yet we also detected a third category of phenotypes corresponding to ‘heterozygote genotypes’. The identification of this third type helps explain colour variation previously observed within each morph (Gill, 1973) and provides a different interpretation of dominance relationships among alleles than recent work based on overall back colour only (Bourgeois et al., 2017). Furthermore, some of the plumage regions were associated with various factors (e.g. light patches related to age, sex, condition) but not to the genomic region that is tightly linked to overall colour, generating unique phenotypes that varied in both the tone of the brown/grey coloration and size and coloration of light patches. A useful next step will be to determine whether general overall coloration, or variation in multiple areas, and/or their covariation, represent structural units (Hebets & Papaj, 2005; Hegyi et al., 2015) with regards to selection. More generally, a focus on continuous quantification of colour may be helpful to describe complex multiple or multicomponent signalling, selection on colour traits and their evolutionary consequences in colour polymorphic systems.

## AUTHOR CONTRIBUTIONS

B.M. and C.T. initiated the project and coordinated the long‐term collection of field data and samples; M.C.M., A.S.C., B.M., Y.B. and C.T. conceived the study and designed the experiments. Molecular data were obtained by M.C.M and L.S., with contributions from M.H.; M.C.M. analysed the data, with contributions from A.S.C. and Y.B.; and M.C.M. and A.S.C. wrote the first draft with input from all authors. All authors gave their final approval for publication.

## CONFLICT OF INTEREST

The authors have no conflict of interest to declare.

## Supporting information


Appendix S1

## Data Availability

The data that support the findings of this study are openly available in figshare at http://doi.org/10.6084/m9, reference number 19723447 ;Sample of birds pictures are available in figshare at: https://doi.org/10.6084/m9.figshare.20806009.v1; Repeatability data for color score analysis are available in figshare at https://doi.org/10.6084/m9.figshare.21411693.v1
