# Supplementary material for: Beyond morphs: Inter‐individual colour variation despite strong genetic determinism of colour morphs in a wild bird
Source: J Evol Biol. 2022 Dec 9;36(1):82–94. doi: 10.1111/jeb.14124 (PMC10107803; doi:10.1111/jeb.14124)
Supplement: Supplementary file 1 — Appendix S1 [file JEB-36-82-s001.docx]

**Supplementary Tables:**

**Table S1**: PCR conditions based on SNP genotyping for two markers associated with back coloration (marker 661151 and 289045), and for a marker used for sex identification (MSZ1R/2550F)

| Tests | Marker name | Forward primer | Reverse primer | PCR conditions |
| --- | --- | --- | --- | --- |
| Plumage color factors | 661151 | CAGGGATGAAAAAGAAATGCG/A | TGGCCAAGCCTAGA  GCTGTG | Touch down 60°C for 25 cycles |
|  | 289045 | TGTTTTACTTTTCCTCCG/A | CCCTTCTTGGGCAA  CATGCAGG | Touch down 57°C for 25 cycles |
| Sex | MSZ1R/2550F | ATCCATCAAGTCTCTAAAGAG | GTTACTGATTCGT  CTACGAGA | Touch down 50°C for 38 cycles |

**Table S2**: Indices used by the package *NbClust* to determine the best number of cluster (from 2 to 10) that emerges from our data (raw coloration score of the back, crown, auricular and flanks), and their corresponding references.


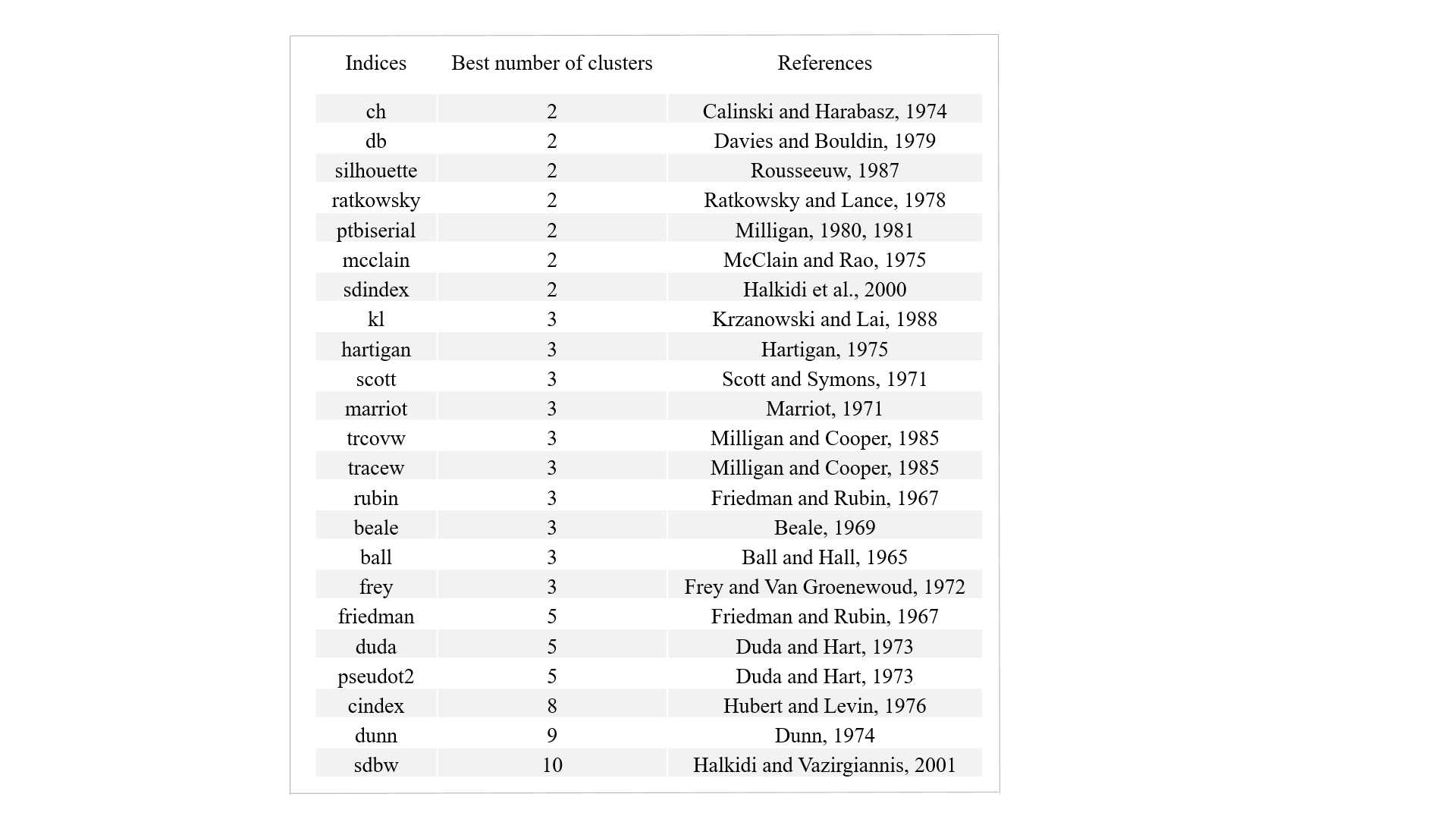


**Table S3**: Relationship between components of bib patches and individual characteristics including age, sex, and condition. Each line represents one model with the bib patch components as the dependent variable and statistics for each of the three predictor variables.


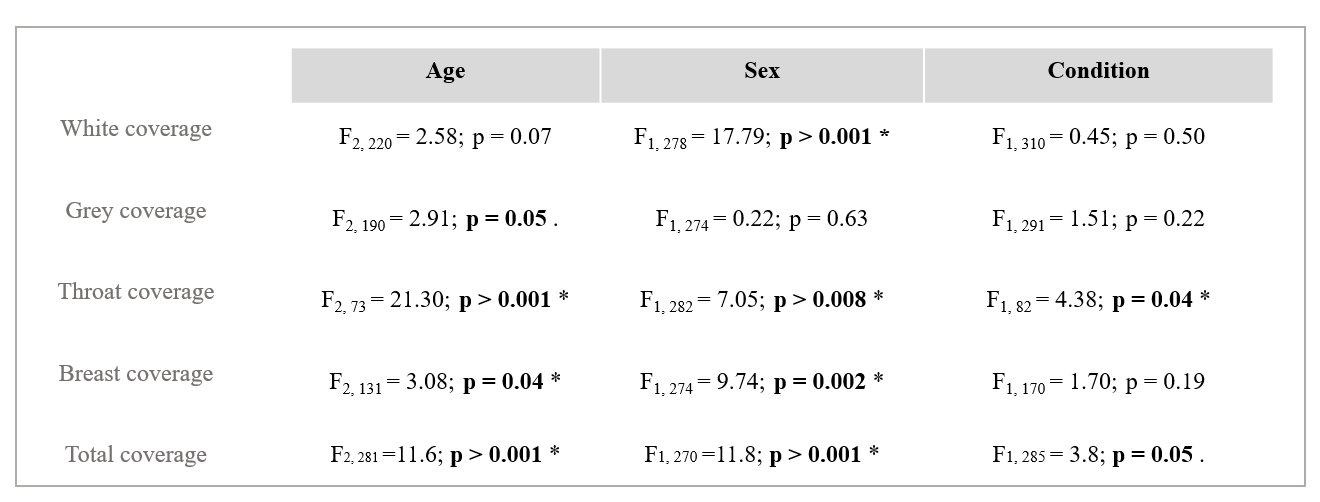


**Table S4:** Relationship between raw coloration score for individual plumage areas (back, crown, auricular, flank, breast, moustache throat) or patch size (rump) and genotypes either at both SNPs markers combined or at marker 661151 and marker 289045 independently. Each result represents one statistical model with the coloration score as the dependent variable and statistics for each of the three predictor variables.


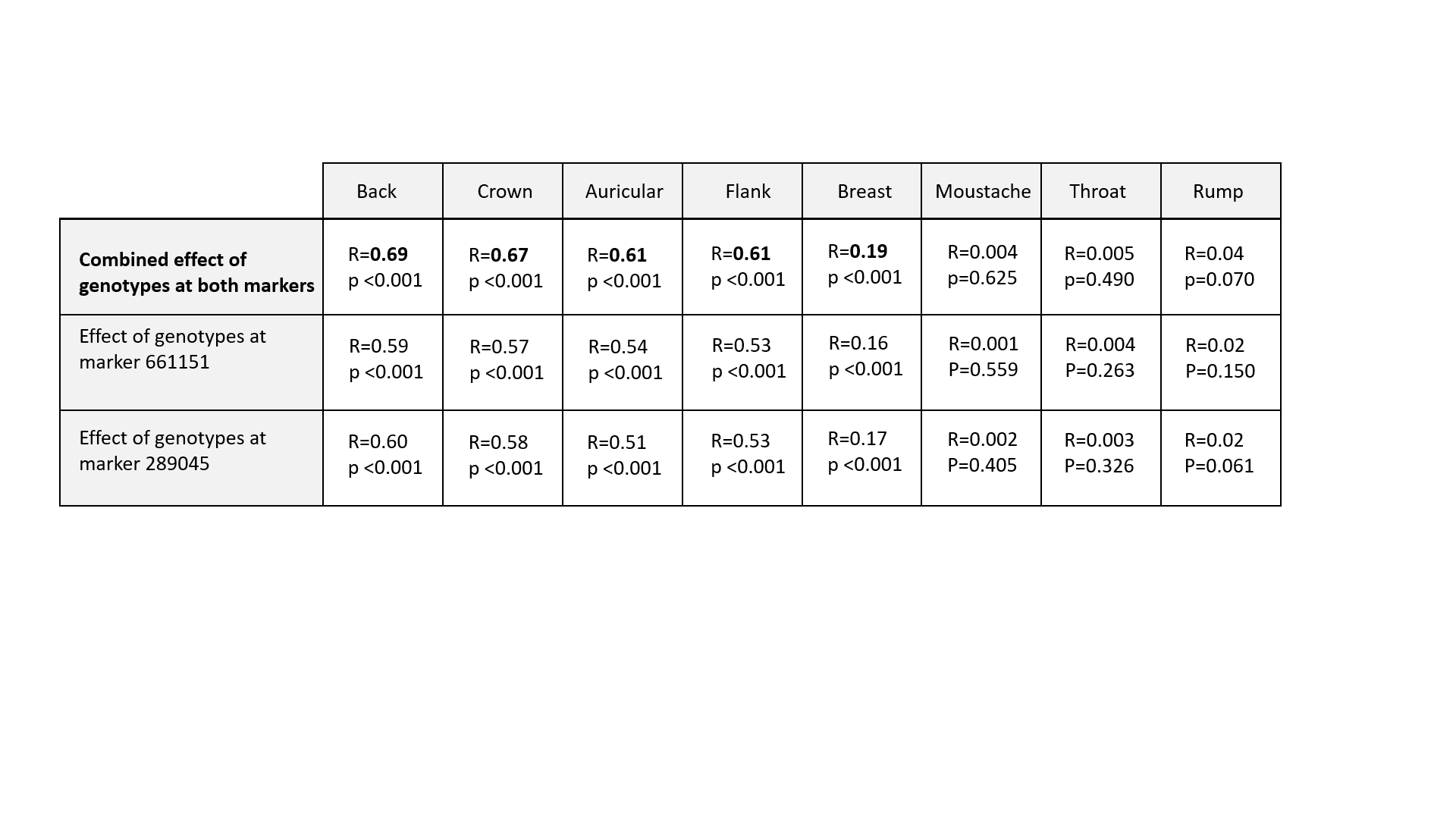


**Supplementary Figures:**


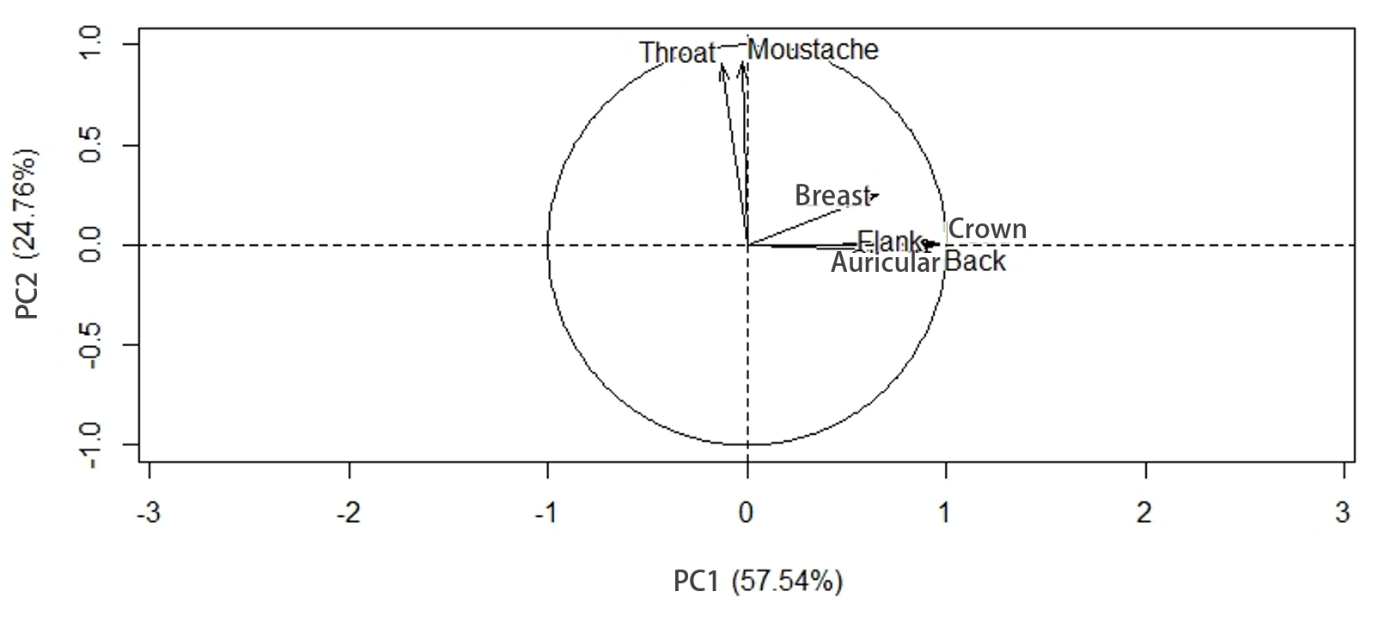


**Fig.S1**: Factors loadings on the first two principal component axes from a PCA including 7 variables (coloration scores on seven body parts). Back, crown, auricular and flank coloration loaded heavily on the first axis (PC1) that explained 57.54% of the variation. Moustache and throat coloration loaded on the second axis (PC2) that explained 24.76% of the variation. Breast color loaded on both axes, although more so on the first than the second.


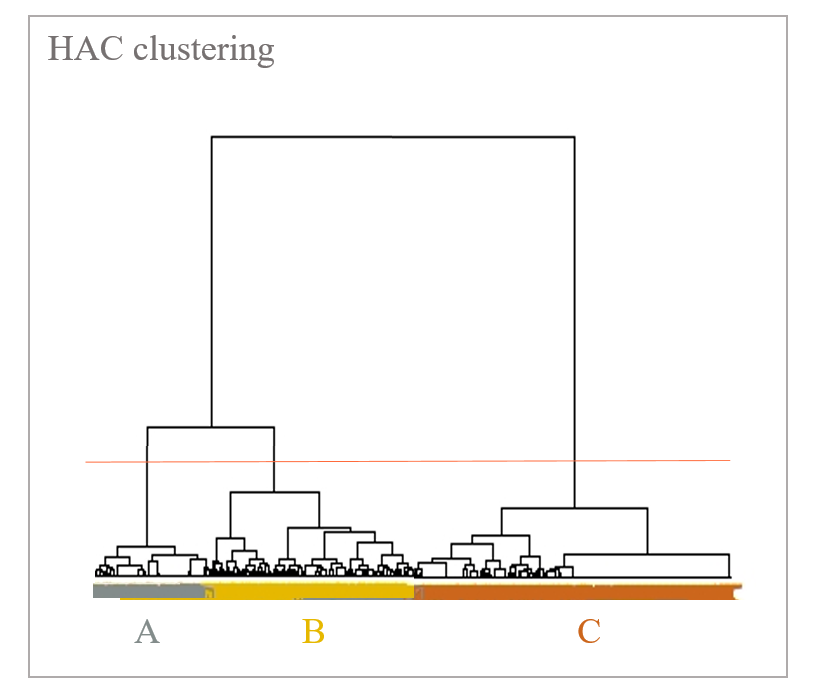


**Fig.S2:** Dendrogram performed through HAC analysis. The red line represents the threshold number of groups chosen using 24 indices proposed by the R package NbClust. The three clusters correspond to grey phenotypes (A), intermediate phenotypes (B) and to brown phenotypes (C).


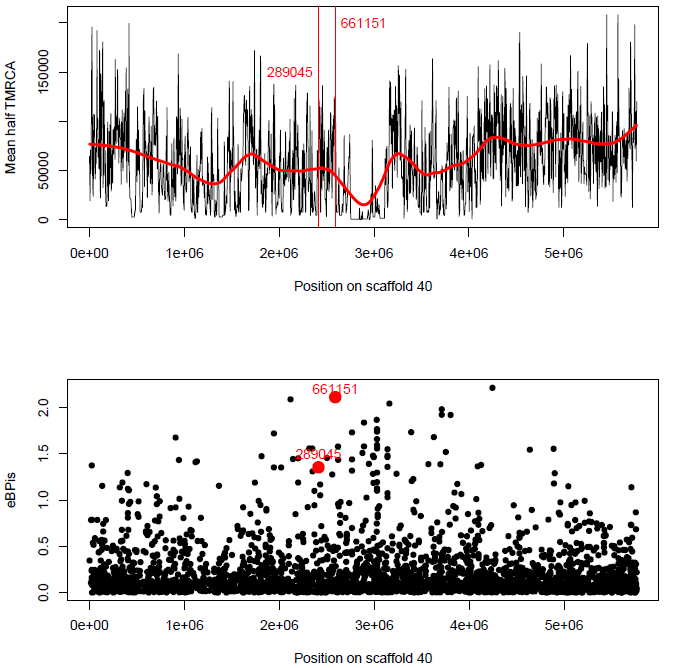


**Fig.S3**: Empirical Bayesian P-value of association (*eBPis*) between allele frequencies and Brown/Grey phenotype (clusters A/B v C in the present study, see Fig. S2). Data were obtained from a pooled RAD-sequencing experiment. Scaffold 40 originates from *Zosterops lateralis* reference genome (available at http://m.ensembl.org/Zosterops_lateralis_melanops/Info/Annotation#page_nav) and aligns to Zebra Finch chromosome 1. Both markers are situated near 96.5Mb on Zebra Finch chromosome 1 (marker 661151 position is 1:96396959-96397336 and marker 289045 position is 1:96580747-96581102). All statistics and data taken from Bourgeois et al. 2017, *RSOS **.

* Bourgeois, Y., Milá, B., Thébaud, C., Delahaie, B., Gautier, M., Lhuillier, E., … Holota, H. (2017). A novel locus on chromosome 1 underlies the evolution of a melanic plumage polymorphism in a wild songbird. *Royal Society Open Science*, *4*, 1–14. <https://doi.org/10.1098/rsos.160805>


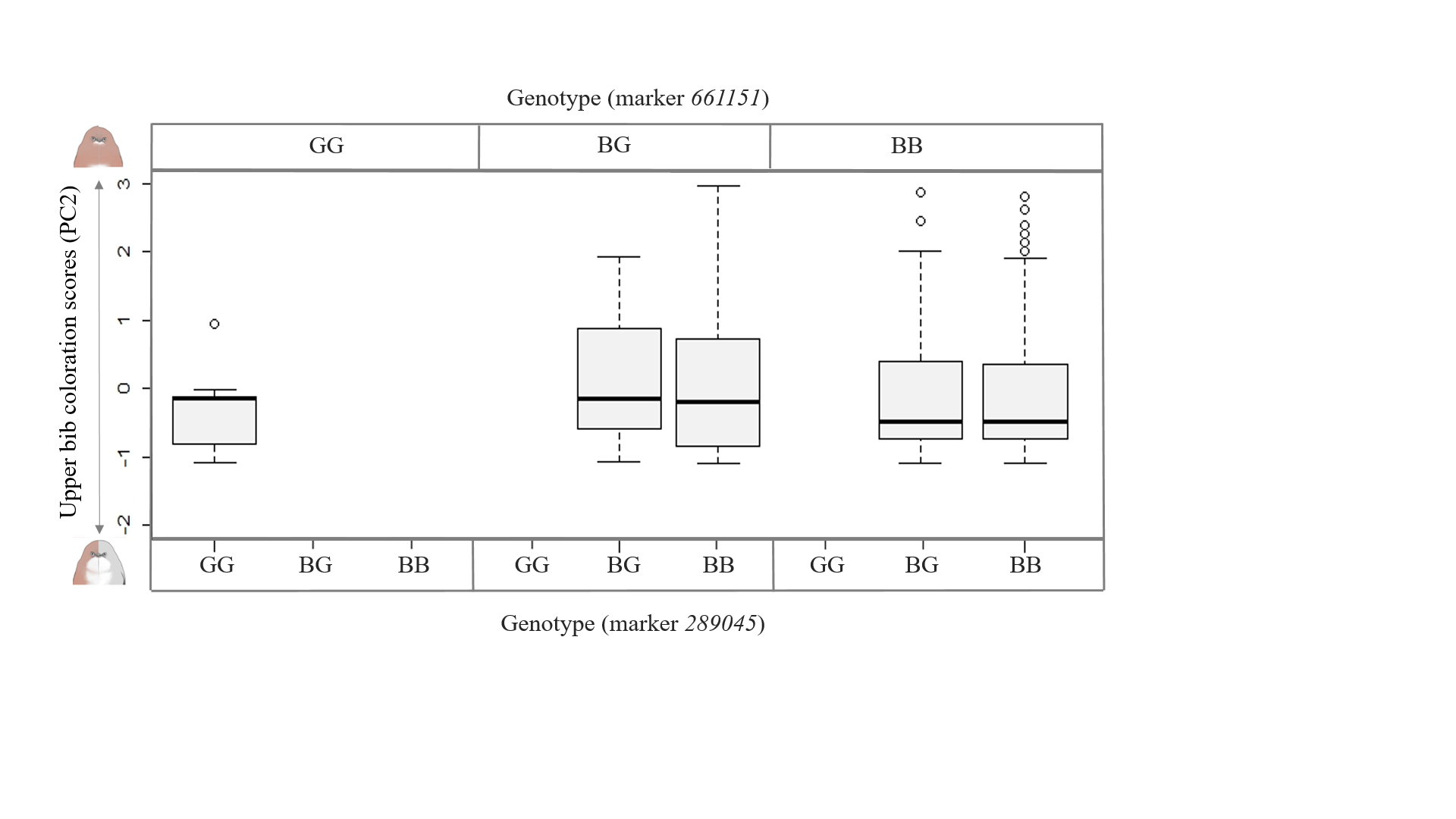


**Fig.S4:** Comparisons of the upper bib coloration score (PC2) between the five observed genotypic classes at both markers.
